# Effects of Compound Elicitors on the Biosynthesis of Triterpenoids and Activity of Defense Enzymes from *Inonotus hispidus* (Basidiomycetes)

**DOI:** 10.3390/molecules27092618

**Published:** 2022-04-19

**Authors:** Jiao Zhou, Xinyue Lin, Shuangshuang Liu, Zhanbin Wang, Dongchao Liu, Yonghong Huo, Dehai Li

**Affiliations:** School of Forestry, Northeast Forestry University, Harbin 150040, China; z1308657484@163.com (J.Z.); linxy_0214@163.com (X.L.); x15990016154@163.com (S.L.); wangzbchina@163.com (Z.W.); ldc8234600892022@163.com (D.L.); hyh1767820626@163.com (Y.H.)

**Keywords:** *Inonotus hispidus*, combination of elicitors, defense enzyme, triterpenoid

## Abstract

*Inonotus hispidus* has various health-promoting activities, such as anticancer effects and immune-stimulating activity. The commercialization of valuable plant triterpenoids faces major challenges, including low abundance in natural hosts and costly downstream purification procedures. In this work, orthogonal design was used to compound methyl jasmonate (MeJA), salicylic acid (SA), oleic acid, and Cu^2+^, and the effects of combinations on the total triterpenes biosynthesized were studied. The optimal combination was screened out and its effect on the activity of PAL, CAT, and SOD was studied. The optimal concentration of oleic acid was 2% when MeJA was 100 mol/L, and the total triterpenoid content and mycelia production were 3.918 g and 85.17 mg/g, respectively. MeJA treatment induced oxidative stress, and at the same time increased the activity of related defense enzymes. Oleic acid is thought to regulate cell permeability by recombining cell membranes. It promotes the material exchange process between cells and the environment without affecting cell growth. When oleic acid was used in combination with MeJA, a synergistic effect on triterpene production was observed. In conclusion, our findings provide a strategy for triterpenoid enrichment of *I. hispidus*.

## 1. Introduction

The shaggy bracket fungus (*Inonotus hispidus*) is a facultative saprophyte that is widely distributed in northern China [1]. It is mainly a parasite that exists on deciduous trees such as ash and mulberry [2]. Its sclerotia are responsible for anticancer effects, immune-stimulating activity, and a dual effect on tyrosinase. Among the bioactive metabolites of *I. hispidus*, triterpenoids are the most well-known secondary metabolites, and are one of the most important classes of natural products.

Triterpenoids are mostly terpenoids consisting of 30 carbon atoms. Based on the ‘isoprene rule,’ most triterpenoids are considered natural products formed by the condensation of six isoprene units [3]. Triterpenoids have a wide range of industrial applications in the food and cosmetic sectors, as well as significant potential applications in the pharmaceutical industry [4]. For instance, phytosterols are used to lower blood low-density lipoprotein cholesterol; ergosterol is used to synthesize vitamin D2 (ergocalciferol); and the plant triterpene betulinic acid has shown promise for the treatment of human immunodeficiency virus and certain cancers in animal models [5]. In particular, triterpenoids isolated from *I. hispidus* demonstrate strong biological activities, such as allelopathy, antifungal, and anti-insect activities [6]. Despite their huge pharmaceutical potential, triterpenoids are the least engineered class of terpenoids. Additional sustainable production platforms are needed to resolve the difficulties in producing and purifying large quantities of industrially relevant triterpenes from their natural sources coupled with environmental concerns.

Elicitors are biological, chemical and physical external factors able to switch on enzymatic responses to biotic or abiotic stresses as well as signaling pathways, leading to the accumulation of secondary metabolites [7]. Introducing elicitors can be an effective method to increase the accumulation of triterpenoids. Jasmonates are common lipid-derived compounds that function as signaling molecules in a plant’s responses to abiotic and biotic stresses, and also function in plant growth and development. A previous study reported the effect of methyl jasmonate (MeJA) on the growth of Aspergillus parasiticus and the aflatoxin B1 (AFB1) output in yeast extract and sucrose medium when added at three different concentrations; namely, MeJA at 10^−4^ and 10^−6^ increased the production of secondary metabolite AFB1 by 141.6% and 212.7%, respectively, compared with the control, whereas treatment with 10^−2^ M MeJA inhibited mycelial growth and AFB1 production [8]. Additionally, MeJA and oleic acid are the most effective elicitors of some metabolites, including triterpenoids. Oleic acid has been used as a stimulatory agent to increase the production of triterpenes and promote the growth of mycelia [9]. In addition, salicylic acid (SA), an endogenous plant growth hormone, plays a pivotal role in regulating stress response and developmental processes in many plants [10]. At present, Zn^2+^ and Cu^2+^ have been extensively studied and applied in the growth and metabolism of edible fungi, such as in the composition of enzymes, activation of enzymes, maintenance of the structure of edible fungal cells, and promotion of the growth of edible fungi. In the process of fermenting the Poria cocos mushroom, the addition of ions can increase the yield of triterpenes [11]. Currently, there is sufficient information available to understand the stimulatory effect of elicitors on the accumulation of triterpenoids [12]. However, few reports are available to describe the different effects of compound elicitors on triterpenoids.

Plants are equipped with an arsenal of adaptive strategies to endure harsh conditions [13]. The chief strategy includes the initiation of systemic signals from an area under stress to an unstressed region, which consequently alerts and activates defense mechanisms or increases resilience arising from signal transducers, including reactive oxygen species (ROS). External environmental stress, such as that caused by exogenous application of an inducer, triggers a burst of ROS, which mainly includes hydrogen peroxide (H_2_O_2_), hydroxyl free radical (·OH), superoxide anion (O_2_^−^), and singlet oxygen (^1^O_2_). ROS are involved in a highly coordinated manner to regulate stress [14]. They activate antioxidants, kinases, defense genes, influx of Ca^2+^ ions, and protein phosphorylation, and increase the synthesis of plant hormones such as salicylic acid, jasmonic acid, and ethylene. In the case of biotic stress, ROS elicit early defense responses such as the synthesis of plant defensive secondary metabolites, and directly or indirectly activate the expression of resistance genes and defense genes to restrict the invasion, multiplication, or spread of pathogens in plant cells [14,15]. However, the high levels of ROS destroy macromolecular substances in plants and are toxic to cells. Therefore, when ROS are produced in large quantities, the amounts of some antioxidant enzymes such as superoxide dismutase (SOD), catalase (CAT), and phenylalanine ammonia lyase (PAL) also increase with the accumulation of ROS to clear and protect the plants [16].

In this work, orthogonal design was used to compound MeJA, SA, oleic acid, and Cu^2+^, and the effects of these combinations on the biosynthesis number of total triterpenes in *I. hispidus* were studied using the dry weight of mycelia and the production of triterpenes as indexes. The optimal elicitor combinations were selected to promote triterpenoid synthesis and mycelial growth. The elicitor may promote the production of triterpenes by activating the cell defense mechanisms. Therefore, the effect of the optimal elicitor combination on activities of PAL, CAT, and SOD was also studied, which provided a reference for the production and utilization of triterpenoids from *I. hispidus*.

## 2. Materials and Methods

### 2.1. Materials and Chemicals

*I. hispidus* was procured from the forest conservation laboratory of Northeast Forestry University (Harbin, Heilongjiang, China) in the northeastern part of China, situated at latitude 45.7186° N and longitude 126.63237° E. The samples were grown under the same environmental conditions in December 2020 and were stored at 4 °C until further experiments.

MeJA, SA, botulin and vanillic aldehyde were purchased from Sigma-Aldrich (St. Louis, MO, USA). Oleic acid was purchased from Aladdin Co. (Shanghai, China). PAL, CAT and SOD assay kits were purchased from Jiancheng Bioengineering Institute (Nanjing, China). All other chemicals and reagents used in this study were analytical grade.

### 2.2. Strain Cultivation

Cultivation of strains was performed in accordance with the methods previously described by Kang et al. Firstly, the slant strains were inoculated on PDA plates at 4 ℃ and incubated at 28 ℃ for 10 d. Three 12 mm diameter mycelial slices were cut and inoculated in PDA liquid medium. The activated strains were homogenized in a sterile environment, then 8% strain solutions were inoculated in 300 mL potato dextrose broth (pH 7.0, autoclaved at 121 °C for 30 min). To assure stability, each inoculated fungus was derived from the same batch. The culture media were placed on a rotary shaker at 125 rpm and 26 °C for 10 days of growth.

### 2.3. Assay of Mycelial Biomass

As the biotransformation process ended, the fermentation broth and the cultured mycelia were separated using a centrifuge at 12,000× *g* for 10 min. The mycelia were washed three times with distilled water and dehydrated to a constant weight by freeze-drying. Then, the dry weight of the mycelia was measured.

### 2.4. Extraction and Determination of Triterpenoids

Construction of a standard curve was conducted in accordance with the method described by Xu, with some minor modifications [17]. First, 5 mg of a botulin standard was dissolved in 50 mL of absolute ethyl alcohol to obtain a standard stock solution (0.1 mg/mL). Then, 0.10, 0.20, 0.40, 0.60, 0.80 and 1.00 mL of standard solution were separately added to 5 mL volumetric flasks (6 flasks in total). After evaporation to dryness using a 100 °C water bath, 0.2 mL of newly prepared 50 g/L vanillin–glacial acetic acid solution and 0.8 mL of perchloric acid were added and shaken well. The solutions were placed in a water bath at 70 °C for 15 min, and then maintained at room temperature for 3–5 min, adjusted to 5 mL with ethyl acetate, and shaken well. Absorbance was measured at 551 nm against the control (composed of 0.2 mL of newly prepared 50 g/L vanillin–glacial acetic acid solution, 0.8 mL of perchloric acid and 4 mL of ethyl acetate). A standard curve was constructed with the weight of botulinal (μg) as the horizontal axis and the OD value as the vertical axis. The linear regression equation was as follows: Y = 0.0052x + 0.0027 (R^2^ = 0.9997).

The determination of triterpenoid content was carried out in accordance with the method described by Xu and Wang, with minor modifications [18]. The dried mycelia were crushed and passed through a 60-mesh sieve, then mixed with 72% (*v*/*v*) ethyl alcohol so that the solid-to-liquid ratio was 1:69. The triterpenoids were ultrasonically extracted using an extraction time of 31 min and extraction power of 210 W. After removal of the mycelia by centrifugation at 12,000× *g* for 10 min, the supernatants were collected.

Finally, 0.2 mL supernatant was measured in accordance with the preparation method of the standard curve. The content of triterpenoids was determined according to the following formula:

(1) $$\mathrm{Content}\;\mathrm{of}\;\mathrm{triterpenoids}\;\left(\mathrm{mg}/g\right)=\frac{\left(Y-0.0027\right){\;N\_10}^{-3}}{0.0052\;m}.$$

### 2.5. Combination of Elicitors

#### 2.5.1. Combination of SA and MeJA

First, 0.100 g of SA was dissolved in 0.2 mL of 95% (*v*/*v*) ethyl alcohol and adjusted to 100 mL with sterile water so as to obtain the mother liquor (1 mg/mL). The mother liquor was filtered with a 0.45 μm filter membrane to remove bacteria, then it was added to different culture media on the 0th day, with final concentrations of 50, 100, and 150 mg/L. MeJA was added to sterile water to produce three solutions with final concentrations of 50, 100, and 150 μmol/L. Tween-20 (0.2% *v*/*v* of each solution) was added as a co-solvent. The solutions were sterile-filtered with a 0.22 μm filter membrane, and after 6 days of fermentation, different concentrations of MeJA solution were added to the medium at 2 μL/mL. Sterile water, 100 mg/L SA, and 50 μmol/L MeJA were used as control groups. All samples were analyzed in triplicate. Fermentation was conducted in accordance with the method described in Section 2.2. The dry weight of the mycelia and the content of triterpenoids were determined after fermentation.

#### 2.5.2. Combination of MeJA and Oleic Acid

Sterilized oleic acid at 3%, 4%, and 5% (*v*/*v*) was added to different culture media on the 0th day, and after 6 days of fermentation, three different concentrations of MeJA solutions (50 μmol/L, 100 μmol/L, and 150 μmol/L, respectively) were added at 2 μL per mL to the culture media in a sterile environment. Sterile water, 3% oleic acid and 50 μmol/L MeJA were used as control groups. All samples were analyzed in triplicate. Fermentation was conducted in accordance with the method described in Section 2.2. The dry weight of the mycelia and amount of triterpenoids were determined after fermentation.

#### 2.5.3. Combination of SA and Oleic Acid

Before fermentation, different media were added to final concentrations of 50, 100 and 150 mg/L of SA, while 3% (*v*/*v*), 4% (*v*/*v*) and 5% (*v*/*v*) oleic acid in culture medium were sterilized. Sterile water, 3% oleic acid and 100 mg/L SA were used as control groups, and all samples were analyzed in triplicate. Fermentation was conducted according to the method described in Section 2.2. The dry weight of the mycelia and the amount of triterpenoids were determined after fermentation.

#### 2.5.4. Combination of SA and Cu^2+^

SA was added to different culture media on day 0, with final concentrations of 50, 100 and 150 mg/L. Then, distilled water was added to 0.1 g copper sulphate and adjusted to 100 mL, which was sterilized at 121 °C for 30 min and added to the culture media on day 3. Copper sulphate was added to the culture media so that the final concentrations were 50, 100, and 150 μmol/L. respectively. Sterile water, 100 mg/L SA, and 100 μmol/L Cu^2+^ were used as control groups. All samples were analyzed in triplicate. Fermentation was conducted in accordance with the method described in Section 2.2. The dry weight of mycelia and the amount of triterpenoids were determined after fermentation.

#### 2.5.5. Combination of MeJA and Cu^2+^

Copper sulphate was added to different culture media on day 3, with final concentrations of 50, 100, and 150 μmol/L. After 6 days of fermentation, three different concentrations of MeJA solutions (50 μmol/L, 100 μmol/L, and 150 μmol/L) were added to each of the three-culture media in a sterile environment at 2 μL/mL. Sterile water, 50 μmol/L MeJA, and 100 μmol/L Cu^2+^ were used as control groups. All samples were analyzed in triplicate. Fermentation was conducted in accordance with the method described in Section 2.2. The dry weight of the mycelia and the content of triterpenoids were determined after fermentation.

#### 2.5.6. Combination of Oleic Acid and Cu^2+^

Different volumes of oleic acid (3%, 4%, and 5%) stock solution were added to the prepared culture media, and this was considered day 0 feeding time. Different concentrations of copper sulphate were supplemented on day 3 of the cell culture to obtain final concentrations of 50, 100, and 150 μmol/sterile water, and 3% oleic acid and 100 μmol/L Cu^2+^ were used as control groups. All samples were analyzed in triplicate. Fermentation was conducted in accordance with the method described in Section 2.2. The dry weight of the mycelia and the content of triterpenoids were determined after fermentation.

#### 2.5.7. Testing for Optimal Elicitor Compounds

Through the above six groups of experiments, the optimal elicitor combination was determined based on the number of induced hyphae and total triterpene content. Furthermore, the effect of the optimal elicitor combinations on activities of defensive enzymes was also studied.

### 2.6. Effect of Compound Elicitors on Defensive Enzymatic Activities

#### 2.6.1. Determination of SOD Activity

To measure SOD activity, four different groups were used: (i) the control group, (ii) 2% oleic acid group, (iii) 100 μmol/L MeJA group and (iv) combination of 100 μmol/L MeJA and 3% oleic acid group. The activity was determined using a SOD assay kit following the manufacturer’s protocols.

#### 2.6.2. Determination of CAT Activity

To measure CAT activity, four different groups were used: (i) the control group, (ii) 2% oleic acid group, (iii) 100 μmol/L MeJA group and (iv) combination of 100 μmol/L MeJA and 3% oleic acid group. The activity was determined using a CAT assay kit, following the manufacturer’s protocols.

#### 2.6.3. Determination of PAL Activity

To measure PAL activity, four different groups were used: (i) the control group, (ii) 2% oleic acid group, (iii) 100 μmol/L MeJA group and (iv) combination of 100 μmol/L MeJA and 3% oleic acid group. The activity was determined using a PAL assay kit following the manufacturer’s protocols.

### 2.7. Statistical Analysis

Experimental data are given as the mean ± standard deviation with three replications. Charts were processed using Origin 9.0 software, and significance analyses were performed with SPSS 21.0 software. Different letters in the figures indicate a significant difference (*p* < 0.05), while the same letters indicate a nonsignificant difference (*p* > 0.05). The differences between the amounts of mycelia are represented by A, B, and C, and the differences between the amounts of triterpenoids are represented by D, E, and F.

## 3. Results

### 3.1. Effects of Different Compound Elicitors on the Triterpenoid and Mycelial Content of I. hispidus

#### 3.1.1. Effects of Induction by SA and MeJA on the Biosynthesis of Triterpenoids

**Figure 1:**
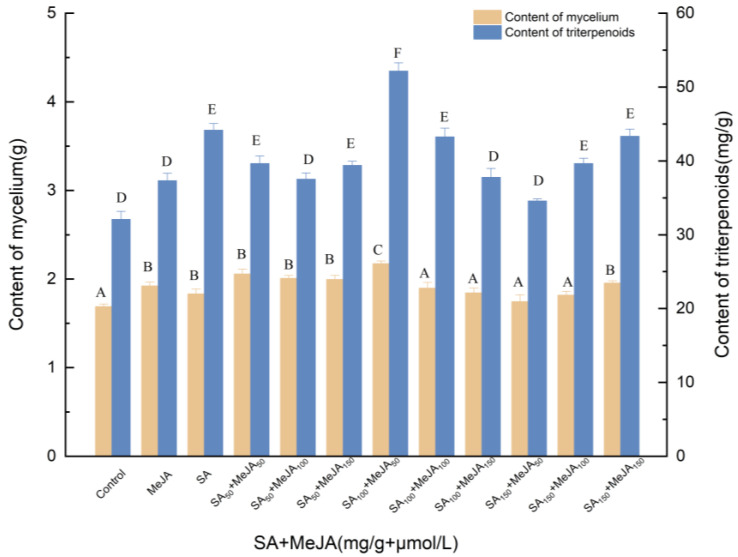
Effect of SA and MeJA on the amounts of mycelia and triterpenoids. Control, sterile water; MeJA, 50 μmol/L; MeJA_50_, MeJA_100_, MeJA_150_, the addition of MeJA was 50, 100, 150 μmol/L, after 6 days of fermentation, MeJA solution were added to the medium at 2 μL per mL; SA, 100 mg/L, SA_50_, SA_100_, SA_150_, the addition of SA was 50, 100, 150 μmol/L, SA was added to different culture media on day 0; +, compound.

To explore the effect of exogenous factors on the production of triterpenes, different concentrations of SA (50, 100, 100 and 150 mg/g) were added to the liquid medium on day 0, and MeJA (50, 100, and 150 μmol/L) was added on day 6. As shown in Figure 1, there were significant differences (*p* < 0.05) in triterpene biosynthesis between the 12 treated control groups and the different experimental groups. Through the analysis, a synergistic effect between SA and MeJA was noted. 

The amounts of triterpenes and mycelia of *I. hispidus* were significantly higher (*p* < 0.05) than those of the control. Because the effect of SA was greater (*p* < 0.05) than that of MeJA, the concentration of SA was considered first. Compared with the control, SA at the concentration of 100 mg/L SA and 50 μmol/L MeJA increased the amount of dried mycelial biomass to 52.163 mg/g and the concentration of triterpenes in fermentation broth to 2.171 g. Compared with SA treatment, mycelia and triterpenes increased by 18.64% and 18.10%, respectively. Compared with MeJA treatment, the mycelia and triterpenes increased by 13.07% and 39.73%, respectively.

#### 3.1.2. Effects of Induction by MeJA and Oleic Acid on the Biosynthesis of Triterpenoids

**Figure 2:**
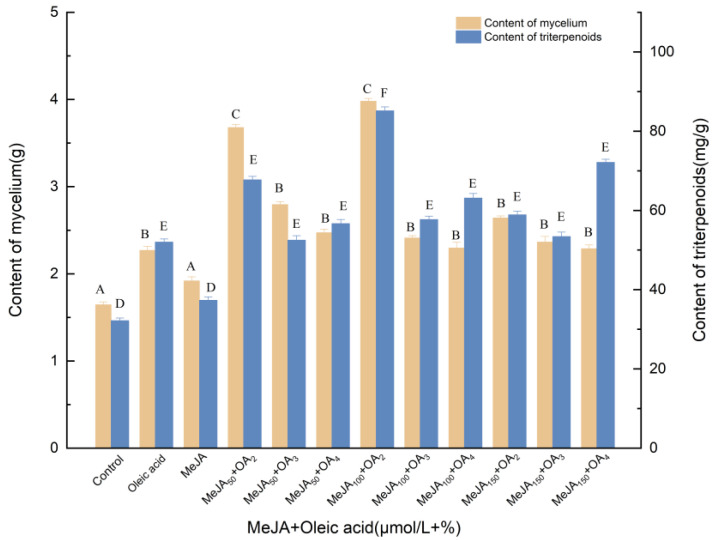
Effect of MeJA and oleic acid on the amounts of mycelia and triterpenoids. Control, sterile water; Oleic acid, 3% (*v*/*v*); OA_2_, OA_3_, OA_4_, the addition of oleic acid was 3% (*v*/*v*), 4% (*v*/*v*) and 5% (*v*/*v*); oleic acid was added to different culture media on day 0; MeJA, 50 μmol/L; MeJA_50_, MeJA_100_, MeJA_150_, the addition of MeJA was 50, 100, 150 μmol/L, after 6 days of fermentation, MeJA solution were added to the medium at 2 μL per mL; +, compound.

Figure 2 shows the concentration-dependent effects of oleic acid and MeJA on the mycelial biomass and triterpene content. Varying amounts of oleic acid and MeJA were added to the culture medium to investigate the influence of concentration and to determine the optimum levels. Oleic acid and MeJA were beneficial for mycelial biomass production, although range analysis showed that the effect of oleic acid was greater (*p* < 0.05) than that of MeJA. Among the 12 tested groups, 2% oleic acid and 100 mol/L MeJA were the most effective in enhancing the mycelial biomass production at all tested concentrations. The mycelial concentration and the concentration of triterpenes significantly increased to 85.17 mg/g and 3.981 g, demonstrating 164.65% and 142.00% increases compared with the control, respectively. As a whole, the optimal concentration of oleic acid can be 2%. When high volume percentages of oleic acid (3%, 4%) were added, the amount of mycelium was significantly reduced, probably due to the excessive concentration of oleic acid covering the surface of the medium, reducing soluble oxygen and thus inhibiting the normal growth of mycelium. Compared with oleic acid treatment, mycelia and triterpenoids increased by 75.37% and 63.69%, respectively. Compared with MeJA treatment, the mycelia and triterpenoids increased by 107.34% and 128.15%, respectively.

#### 3.1.3. Effects of Induction by SA and Oleic Acid on the Biosynthesis of Triterpenoids 

**Figure 3:**
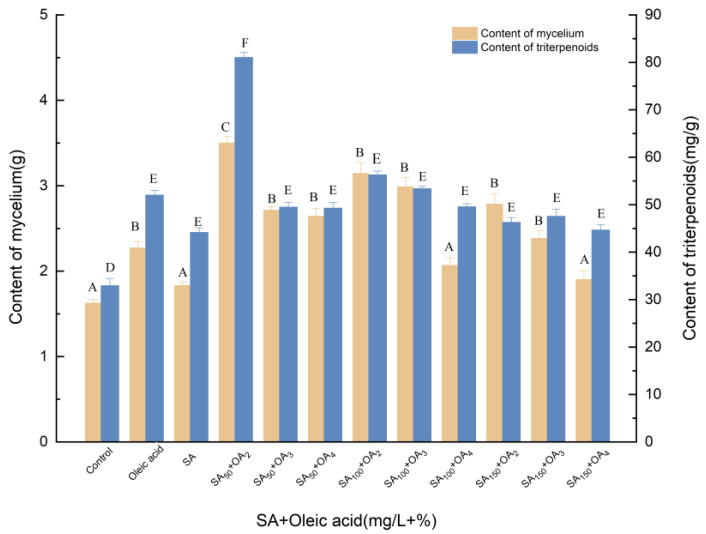
Effect of SA and oleic acid on the amounts of mycelia and triterpenoids. Control, sterile water; Oleic acid, 3% (*v*/*v*); OA_2_, OA_3_, OA_4_, the addition of oleic acid was 3% (*v*/*v*), 4% (*v*/*v*) and 5% (*v*/*v*), oleic acid was added to different culture media on day 0; SA, 100 mg/L; SA_50_, SA_100_, SA_150_, the addition of SA was 50, 100, 150 μmol/L, SA was added to different culture media on day 0; +, compound.

Figure 3 shows the effects of different concentrations of oleic acid and SA on mycelial growth and triterpene production in dry mycelia and fermentation broth. Oleic acid and SA were added to the culture medium upon initial fermentation. Through 12 tested concentrations, it was determined that the optimal concentration of oleic acid was 2%, and when SA was 50 mg/L, the total triterpenoid content and mycelial production were 3.499 g and 81.059 mg/g, respectively, which corresponded to significant (*p* < 0.05) increases of 103.08% and 146.05% compared with the control, respectively. Thus, it was determined that the optimum addition of oleic acid was 2%, and the optimum addition of SA was 50 mg/L. SA is an acid substance widely distributed in plants. It not only participates in many physiological processes, but also is closely related to plant disease resistance. SA can promote the growth of mycelia in the fermentation process of *I. hispidus*. The 50 mg/L SA could participate in the induction of triterpenoid compound synthesis in the early stage. The high concentration of SA was not conducive to the synthesis of triterpenoids in the late stage. Compared with the treatment of oleic acid, the content of mycelium and triterpenoids were increased by 54.14% and 55.79%, resulting in 91.20% and 83.52% higher than the SA group. SA and oleic acid were more effective to improve the biosynthesis of triterpenoids than single induction.

#### 3.1.4. Effects of Induction by SA and Cu^2+^ on the Biosynthesis of Triterpenoids

**Figure 4:**
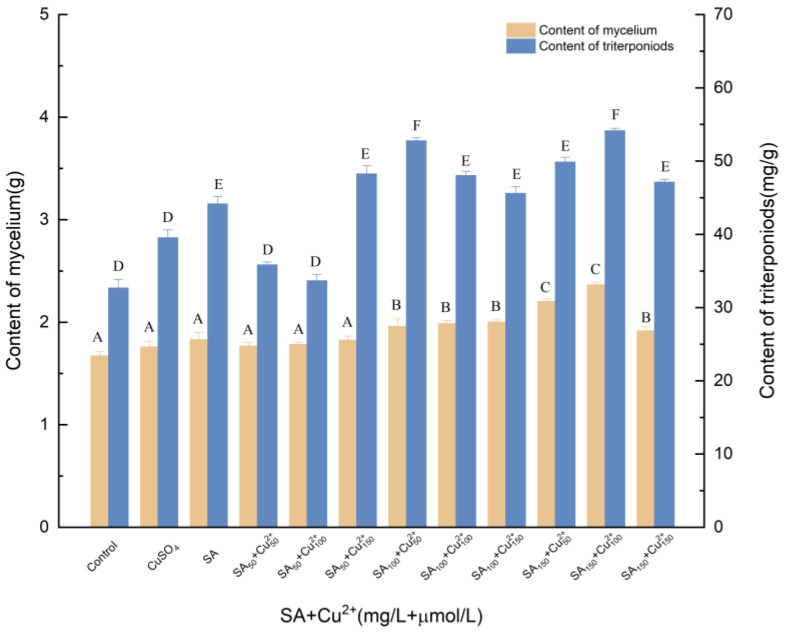
Effect of SA and Cu^2+^ on the amounts of mycelia and triterpenoids. Control, sterile water; CuSO_4_, 100 μmol/L; Cu^2+^_50_, Cu^2+^_100_, Cu^2+^_150_, 50, 100 and 150 μmol/L, supplemented on the 3rd day; SA, 100 mg/L; SA_50_, SA_100_, SA_150_, the addition of SA was 50, 100, 150 μmol/L; SA was added to different culture media on day 0; +, compound.

Figure 4 shows the synergistic effect between SA and Cu^2+^. Treatment with the mixture of Cu^2+^ and SA followed the same trend as that of SA treatment alone, and statistical differences were detected between these two treatments. A synergistic effect of Cu^2+^ and SA on triterpenoid abundance was observed. The addition of 100 μmol/L Cu^2+^ and 150 mg/L SA increased triterpenoid production with maximum stimulation, reaching 2.365 g. Compared with SA treatment, mycelia and triterpenes increased by 28.74% and 22.47%, respectively. Compared with Cu^2+^ treatment, mycelia and triterpenes increased by 34.38% and 36.91%, respectively.

#### 3.1.5. Effects of Induction by MeJA and Cu^2+^ on the Biosynthesis of Triterpenoids

**Figure 5:**
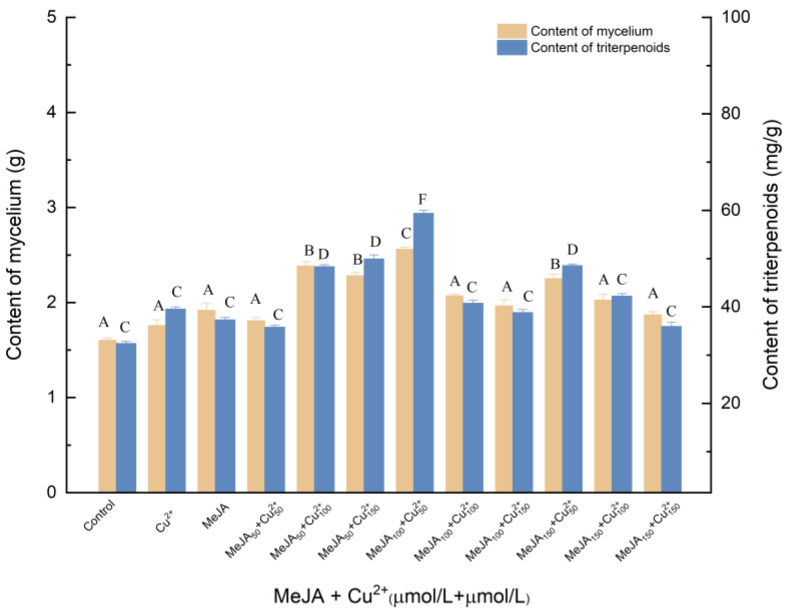
Effect of MeJA and Cu^2+^ on the amounts of mycelia and triterpenoids. Control, sterile water; Cu^2+^, CuSO_4_ 100 μmol/L; Cu^2+^_50_, Cu^2+^_100_, Cu^2+^_150_, 50, 100 and 150 μmol/L, supplemented on the 3rd day; MeJA, 50 μmol/L; MeJA_50_, MeJA_100_, MeJA_150_, the addition of MeJA was 50, 100, 150 μmol/L, after 6 days of fermentation, MeJA solution were added to the medium at 2 μL per mL; +, compound.

Figure 5 presented the effects of the different concentrations of MeJA and Cu^2+^ elicitor on mycelia growth and triterpenes production. As shown in Figure 5, the addition of 100 μmol/L MeJA was the most effective for increasing the yield of triterpenes: when the concentration of Cu^2+^ was 50 μmol/L, triterpenes and mycelia reached 2.561 and 59.407 mg/g, respectively, which was 59.66% and 83.16% higher relative to (*p* < 0.05) the control. It has been found that Cu^2+^ may induce morphological changes in cells, resulting in physiological changes and ultimately affecting the synthesis of triterpenoids. Compared with MeJA treatment, mycelia and triterpenoids increased by 33.89% and 59.14%, respectively. Compared with Cu^2+^ treatment, mycelia and triterpenes increased by 45.51% and 50.16%, respectively.

#### 3.1.6. Effects of Induction by Oleic Acid and Cu^2+^ on the Biosynthesis of Triterpenoids

**Figure 6:**
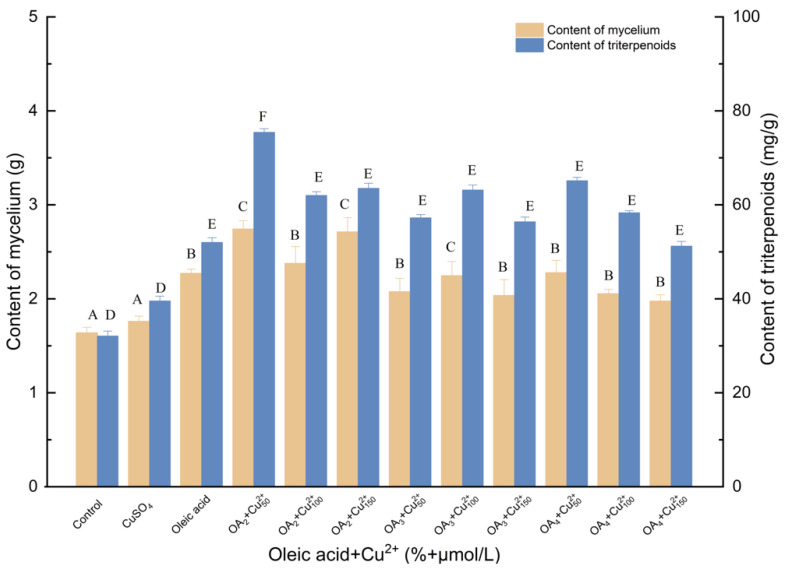
Effect of oleic acid and Cu^2+^ on the amounts of mycelia and triterpenoids. Control, sterile water; CuSO_4_, 100 μmol/L; Cu^2+^_50_, Cu^2+^_100_, Cu^2+^_150_, 50, 100 and 150 μmol/L, supplemented on the 3rd day; Oleic acid, 3% (*v*/*v*); OA_2_, OA_3_, OA_4_, the addition of oleic acid was 3% (*v*/*v*), 4% (*v*/*v*) and 5% (*v*/*v*), oleic acid was added to different culture media on day 0; +, compound.

As shown in Figure 6, when *I. hispidus* was simultaneously elicited with oleic acid and Cu^2+^, the accumulation of triterpenes and mycelia increased. Interestingly, 50 μmol/L Cu^2+^ and 2% oleic acid were the most effective in enhancing mycelial biomass production at all of the tested concentrations. The mycelial and triterpene concentrations significantly increased to 75.45 mg/g and 2.741 g, demonstrating increases of 135.06% and 67.34% compared with the control, respectively. A synergistic effect of both elicitors was evident, as previously indicated. Compared with oleic acid treatment, mycelia and triterpenes increased by 20.75% and 45.01%, respectively. Compared with Cu^2+^ treatment, mycelia and triterpenes increased by 55.74% and 90.72%, respectively.

#### 3.1.7. Determining the Most Optimal Compound Elicitors

**Figure 7:**
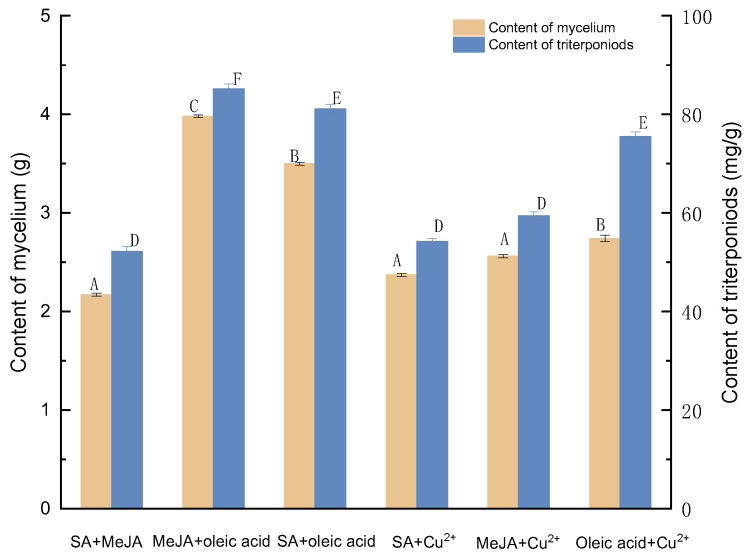
Effect of six compound elicitor combinations on the amounts of mycelia and triterpenoids. SA+MeJA, 100 mg/L SA and 50 μmol/L MeJA; MeJA+oleic acid, 100 mol/L MeJA and 2% oleic acid; SA+oleic acid, 50 mg/g SA and 2% oleic acid; SA+Cu^2+^, 150 mg/g SA and 100 μmol/L Cu^2+^; MeJA+ Cu^2+^, 100 μmol/L MeJA and 50 μmol/L Cu^2^; Oleic acid + Cu^2+^, 2% oleic acid and 50 μmol/L Cu^2+^.

Among the six compound elicitor combinations, the optimal concentration combinations of each group were selected and compared, and the specific results are shown in Figure 7. Oleic acid and MeJA were more effective for increasing triterpene and mycelium yield formation than the remaining five groups. As a consequence, 2% oleic acid and 100 mol/L MeJA were selected for further investigation.

### 3.2. Effect of Oleic Acid and MeJA on Defensive Enzymatic Activities

#### 3.2.1. Effect of Oleic Acid and MeJA on SOD Activity

As shown in Table 1, the combined effect of MeJA and oleic acid increased the SOD activity of *I. hispidus*. On the second day, the SOD activity of the medium containing 2% oleic acid was the highest, reaching 78.70 ± 3.54 U/mg prot, which was 1.42 times higher than that of the medium without elicitor. There was a progressive increase in SOD enzymatic activity in *I. hispidus* treated with 100 μmol/L MeJA on day 7, where SOD enzymatic activity increased by 1.51-fold in *I. hispidus* compared with the control.

SODs provide an initial or first-line defense against toxic ROS [19]. They catalyze the disproportionation of O_2_^–^ free radicals by reducing one radical into H_2_O_2_ and oxidizing another into O_2_, thereby eliminating the risk of production of more toxic ·OH free radicals. In this study, the increase in SOD activity in cells induced by MeJA and oleic acid may be a physiological response to the increase in intracellular ROS, and the SOD enzymatic activity increased with the increase in intracellular ROS [20].

#### 3.2.2. Effect of Oleic Acid and MeJA on CAT Activity

As shown in Table 2, the combined effect of MeJA and oleic acid on intracellular CAT enzymatic activity was obvious, and the induced enzymatic activity was significantly higher than the CAT enzymatic activity in the control group, which did not significantly change and remained at a relatively low level. CAT activity increased with the increase in days and reached the highest level on day 3. The activity of CAT in the medium containing 2% oleic acid was the highest, up to 38.50 ± 3.45 U/mgprot, which was 1.52 times higher than that in the medium without elicitors (*p* < 0.05). After day 6, the SOD activity of the control and culture medium containing 2% oleic acid increased again under the treatment with 100 μmol/L MeJA. On day 8, the highest values were 32.80 ± 3.46 U/mgprot and 37.08 ± 1.97 U/mgprot, which were 1.74 times and 1.97 times higher (*p* < 0.05) than those of the control, respectively. The change in CAT activity may be a protective reaction to the high concentration of H_2_O_2_ caused by the addition of elicitors.

#### 3.2.3. Effect of Oleic Acid and MeJA on PAL Activity

PAL is a key and rate-limiting enzyme in the phenylpropane metabolic pathway [21]. An increase in its activity can increase the amount of lignin, cork, and polyphenols, which then increases the disease resistance or stress resistance. Polyphenols also have a strong free radical scavenging ability and can inhibit cell membrane lipid peroxidation, and therefore, the antioxidant activity of plant cells is also related to the activity of PAL.

The effect of the combination of MeJA and oleic acid on the activity of the PAL enzyme in the cultured cells of *I. hispidus* is shown in Table 3. On day 3, the PAL activity was the highest (*p* < 0.05) in the medium containing 2% oleic acid, up to 40.52 ± 4.42 U/mgprot. In the control and medium containing 2% oleic acid, the activity of PAL decreased with the increase in fermentation days. The highest values on day 7 were 34.62 ± 3.12 U/mg prot and 38.88 ± 3.77 U/mgprot, respectively, which were 1.36 and 1.53 times higher than those of the control.

## 4. Discussion

There is an increasing need for terpene production that is faster and less costly as new industrially relevant molecules are discovered. The purpose of this study was to find the most suitable stimulant for triterpene production by *I. hispidus*. Triterpenes are secondary metabolites with biological specificity and can increase the defensive ability of plants against pathogens [22]. Triterpenes are derived from squalene, which is a molecule produced by the condensation of isoprene phosphate (formed farnesyl pyrophosphate, FPP) synthesized by the valeric acid pathway and/or the plastid 2C-methyl-D-erythritol 4-phosphate (MEP) pathway [23]. In this study, 150 mg/g of SA and 100 μmol/L of Cu^2+^ effectively enhanced the accumulation of triterpenoids and mycelia of *I. hispidus* by 32.49% and 65.68%, respectively, compared with the control. It has been reported that Cu^2+^ is an essential nutrient for biological growth, affects cell growth and secondary metabolism and also causes changes in cell morphology and cell physiology [24]. Therefore, in the early stage of fermentation, adding Cu^2+^ can be used as a nutritional factor to promote mycelial growth and increase the accumulation of triterpenes.

The data indicate that Cu^2+^ is also a cofactor for a variety of proteases involved in a variety of metabolic processes. After SA was added on day 0, a class of biologically specific triterpenoids was produced to increase the ability to defend against pathogens. This is because SA is a phenolic compound with a unique regulatory effect, involving an increase in the activity of antioxidant enzymes in *I. hispidus* that produce H_2_O_2_ through enzymatic reactions, thus promoting triterpenoid synthesis by activating the cell defense mechanism. It has also been reported that the elicitor effect of exogenous SA stimulates the biosynthesis of asiaticoside and hydroxyasiaticoside in *Centella asiatica* cell culture [25].

**Figure 8:**
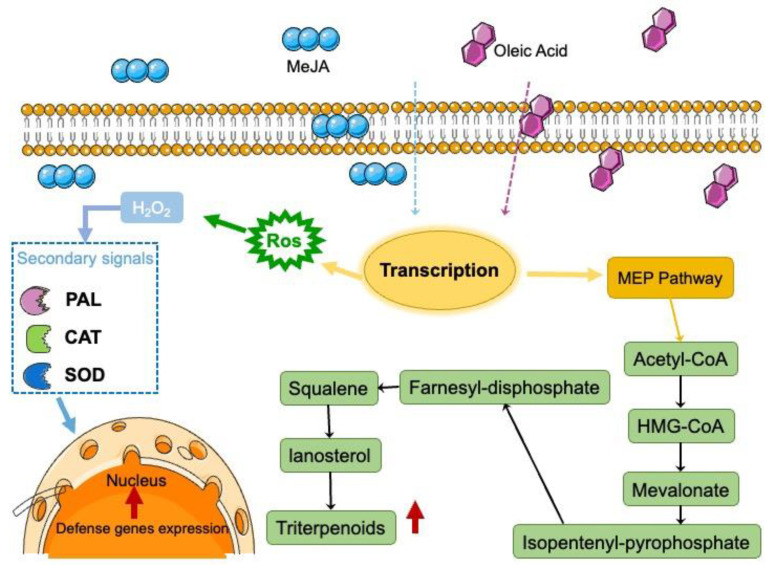
Conceptual diagram of the effects of MeJA and oleic acid on terpene biosynthesis in *I. hispidus*, based on the results of this study. In plants, MeJA triggers a transcriptional rearrangement in the nucleus, which then starts a coordinated transcriptional response leading to an upregulation of the MEP pathway and oxidative stress. The red color indicates an increase and a decrease in metabolite abundance compared with untreated cells. MEP: 2-C-methyl-D-erythritol 4-phosphate; ROS: reactive oxygen species.

There are two possible mechanisms to explain this enhancement. One is attributed to MeJA, which can influence the relative enzymatic activity of secondary metabolites, whereby terpenoids are synthesized via the mevalonate pathway and acetyl-CoA is converted through a series of chemical reactions to 3-hydroxy-3-methylglutaryl-CoA, and then to mevalonate, to isopentenyl-pyrophosphate, to FPP, to squalene, and finally, to lanosterol [26]. During the characterization of these enzymes, a positive correlation has been noted between the amounts of terpenoids produced at different stages and the expression levels of farnesyl pyrophosphate synthase and squalene synthase [27]. MeJA triggers oxidative stress in plants by inducing the production of ROS first in the mitochondria and subsequently in the chloroplasts (Figure 8). Oxidative stress is one of the main causes of MEP pathway rearrangement and upregulation in plant cells, as certain products of this pathway are involved in protection against oxidative stress [28]. MeJA increased the concentration of ROS and induced the activation of related defense enzymes, which activated the upregulation of key enzyme genes of secondary metabolite synthase and finally promoted the synthesis of triterpenes in the cells. A similar report showed that all genes of *Ganoderma lucidum* were upregulated under stimulation [29].

A possible mechanism allowing oleic acid to promote mycelial growth is that unsaturated fatty acids are an important component of membrane phospholipids, and oleic acid is thought to regulate cell permeability by recombining cell membranes [30]. Some studies have reported that stimulatory agents are presumed to mediate cell permeability by reorganizing the cell membrane and/or by directly affecting the synthesis of enzymes involved in the formation of target products [31]. Thus, with oleic acid treatment, *I. hispidus* can absorb a greater amount of nutrients from the liquid medium. Numerous studies have reported that oleic acid can also be used as an oxygen carrier to significantly increase the oxygen concentration and cell membrane permeability in the fermentation system while promoting the process of material exchange between cells and the environment [32].

The inefficient scavenging ability of antioxidants leads to an oxidative explosion in plant cells [33]. To prevent damage to cells caused by excessive ROS, CAT and SOD are the key defense enzymes in the cellular defense system, and can resist the damage to cells caused by external factors. Plants often simultaneously encounter multiple stresses that require discrete antioxidant activity [34]. For example, when *Portulaca oleracea* was subjected to combined heat and drought stress, higher SOD activity was observed. After MeJA and oleic acid treatment, the enzymatic activity of SOD, CAT, and PAL in *Tricholoma hirsutum* was significantly higher than that of the control group [35].

In this study, the SOD activity of the medium containing 2% oleic acid was the highest, reaching 78.70 ± 3.54 μ/mgprot. These results suggest that exogenous MeJA and oleic acid elicitors may induce the production of superoxide anions, followed by the accumulation of H_2_O_2_. As the first line of defense for the removal of ROS in a fermentation system, SOD can disproportionate superoxide anions to form H_2_O_2_, which effectively removes the accumulated ROS in the cells [36]. The addition of MeJA and oleic acid to *I. hispidus* caused the outbreak of CAT activity (Table 2). CAT increases the destruction of H_2_O_2_ by decomposing it into O_2_ and H_2_O, thereby producing benign molecules [37].

After treatment with MeJA and oleic acid, the activity of PAL significantly increased (Table 3). PAL catalyzes phenylalanine to trans-cinnamic acid, which is the first step in the biosynthesis of phenylpropanoid, leading to a diverse group of secondary plant metabolites [38]. Plant antitoxin, p-coumaric acid derivatives and lignin are biosynthesized by PAL. These compounds contribute to plant defense and ultimately promote the synthesis of intracellular triterpenes. In addition, PAL is also actively involved in the biosynthesis of SA, a hormone required for plant defense [38]. These findings suggest that PAL plays an important role in regulating the resistance of ciliated bacteria to exogenous elicitors.

## 5. Conclusions

In summary, we used an orthogonal design to investigate the effects of six combinations of oleic acid, MeJA, SA, and Cu^2+^ on triterpene accumulation in cultures of *I. hispidus*. They all could increase the production of triterpenes and promote the growth of mycelia. The combination of oleic acid and MeJA had the most significant effect. This study showed that the optimal combination triggers oxidative stress in *I. hispidus.* This observation provides evidence that the MEP pathway and the triterpenoid pathway(s) of *I. hispidus* respond to oxidative stress. This finding is likely to spark new research to understand the underlying molecular mechanisms. The detailed strategy for active secretion of triterpenoids was reported in *I. hispidus*, which is very useful for future biotechnological applications such as the production and purification of high-value plant triterpenoids in *I. hispidus*. This approach can be used to up-regulate triterpene pathways in *I. hispidus*. Adequate strategies should be used to enrich the huge pharmaceutical potential. The study showed that MeJA caused oxidative stress in *I. hispidus*. This observation may prove that the MEP and triterpenoid pathways of *I. hispidus* are responsive to oxidative stress. This discovery may trigger new research to understand its underlying molecular mechanism. In *I. hispidus*, we reported the strategy of active secretion of triterpenoids by compound combination, which will be very useful for future biotechnology applications.

## Figures and Tables

**Table 1:** Effect of MeJA and oleic acid on SOD activity in *I. hispidus*.

Day	Activity of SOD(U/mgprot) under the Treatment with Different Elicitors			
	Control	2% of Oleic Acid	100 μmol/L MeJA	2% of Oleic Acid+ 100 μmol/L MeJA
0	48.34 ± 2.20 ^aC^	54.56 ± 2.45 ^aD^	48.34 ± 2.20 ^aC^	54.56 ± 2.45 ^aD^
2	55.27 ± 3.03 ^bC^	78.70 ± 3.54 ^bD^	55.27 ± 3.03 ^bC^	78.70 ± 3.54 ^bD^
3	52.74 ± 2.24 ^bC^	71.86 ± 2.10 ^bD^	52.74 ± 2.24 ^bC^	71.86 ± 2.10 ^bD^
5	47.47 ± 2.78 ^aC^	60.84 ± 2.13 ^cD^	47.47 ± 2.78 ^aC^	60.84 ± 2.13 ^aD^
7	45.60 ± 2.13 ^aC^	52.25 ± 2.03 ^aC^	59.70 ± 3.10 ^cD^	68.69 ± 3.01 ^cF^
8	42.40 ± 1.45 ^cC^	47.40 ± 2.01 ^aC^	56.39 ± 2.89 ^bD^	63.30 ± 2.97 ^aF^
10	42.10 ± 1.98 ^cC^	45.20 ± 2.41 ^aC^	54.60 ± 1.56 ^bD^	60.30 ± 2.45 ^aF^

Each value is the mean of three experiments, and there are significant differences between different lowercase letters in the same column; *p* < 0.05. There are significant differences between different capital letters in the same row; *p* < 0.05. (Lowercase letters: differences in enzymatic activity between different days; Capital letters: differences between different elicitors in inducing enzymatic activity).

**Table 2:** Effect of MeJA and oleic acid on CAT activity (U/mgprot) in *I. hispidus*.

Day	Activity of CAT (U/mgprot) under Treatment with Different Elicitors			
	Control	2% of Oleic Acid	100 μmol/L MeJA	2% of Oleic Acid+ 100 μmol/L MeJA
0	14.57 ± 1.53 ^aC^	16.57 ± 1.41 ^aD^	14.57 ± 1.53 ^aC^	16.57 ± 1.41 ^aC^
2	19.69 ± 2.43 ^aC^	28.33 ± 2.32 ^bD^	19.69 ± 2.43 ^aC^	28.33 ± 2.32 ^bD^
3	25.20 ± 1.50 ^bC^	38.50 ± 3.45 ^cD^	25.20 ± 1.50 ^bC^	38.50 ± 3.45 ^cD^
5	23.20 ± 2.61 ^bC^	29.82 ± 2.67 ^bD^	23.20 ± 2.61 ^bC^	29.82 ± 2.67 ^bD^
7	20.32 ± 1.57 ^bC^	26.80 ± 2.50 ^bD^	28.40 ± 2.56 ^bD^	34.90 ± 1.70 ^bF^
8	18.83 ± 1.49 ^aC^	23.30 ± 1.39 ^aC^	32.80 ± 3.46 ^cD^	37.08 ± 1.97 ^cF^
10	18.16 ± 1.47 ^aC^	22.17 ± 1.40 ^aD^	30.32 ± 2.57 ^cF^	32.32 ± 1.29 ^bF^

Each value is the mean of three experiments, and there are significant differences between different lowercase letters in the same column; *p* < 0.05. There are significant differences between different capital letters in the same row; *p* < 0.05. (Lowercase letters: differences in enzymatic activity between different days; Capital letters: differences between different elicitors in inducing enzymatic activity).

**Table 3:** Effect of MeJA and oleic acid on PAL activity (U/mgprot) in *I. hispidus*.

Day	Activity of PAL (U/mgprot) under Treatment with Different Elicitors			
	Control	2% of Oleic Acid	100 μmol/L MeJA	2% of Oleic Acid+ 100 μmol/L MeJA
0	25.56 ± 3.20 ^aD^	29.56 ± 3.07 ^bD^	25.56 ± 3.20 ^aC^	29.56 ± 3.07 ^aD^
2	30.81 ± 3.65 ^bC^	35.40 ± 4.05 ^cD^	30.81 ± 3.65 ^aC^	35.40 ± 4.05 ^bD^
3	29.07 ± 2.96 ^bC^	40.52 ± 4.42 ^cD^	29.07 ± 2.96 ^aC^	40.52 ± 4.42 ^cD^
5	27.78 ± 2.69 ^bC^	32.64 ± 3.32 ^cD^	27.78 ± 2.69 ^aC^	32.64 ± 3.32 ^bD^
7	25.40 ± 2.21 ^aC^	28.47 ± 3.90 ^bD^	34.62 ± 3.12 ^cF^	38.88 ± 3.77 ^cF^
8	24.20 ± 4.01 ^aC^	26.77 ± 4.07 ^aC^	32.41 ± 3.20 ^bF^	33.45 ± 3.21 ^bF^
10	23.09 ± 3.10 ^aC^	25.07 ± 3.65 ^aC^	29.20 ± 2.01 ^aD^	31.70 ± 2.20 ^aD^

Each value is the mean of three experiments, and there are significant differences between different lowercase letters in the same column; *p* < 0.05. There are significant differences between different capital letters in the same row; *p* < 0.05. (Lowercase letters: differences in enzymatic activity between different days; Capital letters: differences between different elicitors in inducing enzymatic activity).

## Data Availability

The data presented in this study are available in article.
